# Retraction: Mitofusin-2 enhances mitochondrial contact with the endoplasmic reticulum and promotes diabetic cardiomyopathy

**DOI:** 10.3389/fphys.2022.1006583

**Published:** 2022-08-04

**Authors:** Frontiers Editorial Office

The Publisher retracts the cited article.

Following publication, the publisher uncovered evidence that false identities were used in the peer-review process. The assignment of fake reviewers was confirmed by an investigation, conducted in accordance with Frontiers’ policies and the Committee on Publication Ethics (COPE) guidelines.

The investigation also uncovered concerns about the presentation and validity of the data in the article that normally would have led to a rejection. When contacted, the authors failed to provide a data set that adequately supports the reported conclusions.

The authors have not responded to correspondence regarding this retraction.

This retraction was approved by the Chief Editors of Frontiers in Physiology and the Chief Executive Editor of Frontiers.

